# Associations Between Sleep, Appetite, and Food Reward over 6 Months in Black Emerging Adults—Findings from the Sleep, Health Outcomes and Body Weight (SHOW) Pilot Study

**DOI:** 10.3390/nu17142305

**Published:** 2025-07-13

**Authors:** Hannah R. Koch, Jesse N. L. Sims, Stephanie Pickett, Graham Finlayson, Laurie Wideman, Jessica McNeil

**Affiliations:** 1 Department of Kinesiology, University of North Carolina at Greensboro, 1408 Walker Avenue, Greensboro, NC 27412, USA; hrkoch@uncg.edu (H.R.K.); l_widema@uncg.edu (L.W.); 2 Department of Exercise Science and Exercise Physiology, Kent State University, Kent, OH 44242, USA; jsims26@kent.edu; 3 School of Nursing, University of North Carolina at Greensboro, Greensboro, NC 27402, USA; s_picke2@uncg.edu; 4 School of Psychology, University of Leeds, Leeds LS2 9JT, UK; g.s.finlayson@leeds.ac.uk

**Keywords:** sleep, appetite, food reward, emerging adulthood, obesity

## Abstract

**Background/Objectives**: Imposed sleep restriction leads to increased feelings of appetite and hedonic eating behaviors (or food rewards). No study to date has assessed home-based measures of sleep with appetite and food rewards exclusively in Black emerging adults (ages 18–28 years), despite higher risks of short sleep and obesity in this population. We examined associations between 6-month changes in sleep with changes in appetite and food reward in Black emerging adults. **Methods**: Fifteen Black emerging adults (12 females; age, 21 ± 2.5 years; body mass index, 25.7 ± 4.5 kg/m^2^; body fat, 25.8 ± 11.9%) completed two identical 7-day measurement bursts at baseline and 6 months. Sleep (duration, efficiency, and architecture) was captured via 7 days of actigraphy and 2 nights of in-home polysomnography. During a laboratory visit, participants completed appetite measures (desire to eat, hunger, fullness, and prospective food consumption) via visual analog scales before and for 3 h following standard breakfast intake. The food reward for the fat and sweet categories of food was measured before lunch with the Leeds Food Preference Questionnaire. **Results**: Fasting fullness scores decreased from baseline to 6 months (−8.9 mm, *p* < 0.01) despite increases in body weight (2.6 kg, *p* < 0.01) and waist circumference (2.4 cm, *p* = 0.03). Increases in actigraph-measured sleep duration were associated with decreases in fasting desire to eat (r = −0.58, *p* = 0.04). Increases in actigraph-measured sleep efficiency were also associated with decreases in explicit liking for sweet foods (r = −0.60, *p* = 0.03). **Conclusions**: Our findings suggest that improvements in sleep duration and sleep efficiency may lead to decreased feelings of appetite and food reward in Black emerging adults.

## 1. Introduction

Energy intake, a key component of energy balance that may contribute to weight gain and obesity development, is influenced by both homeostatic (i.e., energy need or energy expenditure) and non-homeostatic (i.e., environmental factors, food hedonics, or reward) factors [1]. Although dynamic energy intake is mainly driven by homeostatic factors, non-homeostatic signals can override the homeostatic control of feeding in an obesogenic environment and lead to weight gain via excess energy intake [1]. Emerging adults (approximately ages 18–28 years) have a greater risk of excess weight gain; however, data on potential changes in appetite and food reward in this age group is currently scarce.

Insufficient sleep (i.e., shorter total sleep duration, shorter slow-wave sleep (SWS), and rapid eye movement (REM) sleep durations, and lower sleep efficiency (<85%)) is consistently associated with the risk of obesity and weight gain [2]. Zhu and colleagues [3] conducted a meta-analysis of sleep restriction trials and noted a 13.4 unit increase in subjective hunger compared to habitual sleep duration. Changes in sleep architecture itself may also be related to changes in appetite sensations. Rutters and colleagues [4] reported that greater SWS duration was associated with greater fasting fullness and lower fasting hunger before dinner. Additionally, greater REM sleep duration has been associated with lower post-meal hunger [5] and whole-day hunger scores [6]. Furthermore, sleep restriction appears to enhance food reward. Specifically, sleep restriction has been shown to lead to increased wanting and liking for high-fat foods [5], sweet taste preferences [7], and greater neural activation in the brain’s reward circuitry [8,9]. Conversely, 1.6 h of sleep extension in short sleepers led to a decrease in overall appetite and a lower desire to consume sweet foods [10]. Much of the evidence on short sleep with appetite and food reward stems from laboratory-based sleep studies that induce restrictions or modifications in habitual sleep. In free-living conditions (or home environments), the relationships between sleep with appetite and food reward remain unclear.

Black individuals are at a disproportionately higher risk of obesity [11,12] and insufficient sleep [13,14,15,16] compared to their White counterparts. This may be due, in part, to race-related stressors such as individual- and systemic-level racism and discrimination, as well as unequal access to medical care and health-related resources (Williams & Mohammed, 2009 [17]). Dorling and colleagues [18] also reported no racial differences in energy intake, despite lower self-reported desire to eat and greater feelings of satiety in Black individuals compared to their White counterparts. Yet, the relationship between sleep with appetite and food reward in this population has not been investigated. To address this, we conducted pilot data collection in 15 Black emerging adults (ages 18–28 years), which also assessed the feasibility for a larger-scale study called the Sleep, Health Outcomes and Body Weight (SHOW) study [19]. The purpose of this analysis was to assess whether changes in actigraphy- and polysomnography- (PSG) derived sleep variables are associated with changes in appetite and food reward over a 6-month period in this sample. Based on the evidence from in-laboratory studies [5,6,7,10], we hypothesized that greater insufficient sleep (i.e., shorter sleep duration, shorter SWS and REM sleep durations, and lower sleep efficiency) would be associated with greater increases in subjective appetite measures (i.e., greater feelings of hunger and lower fullness) and wanting for high-fat and high-sweet foods over 6 months.

## 2. Materials and Methods

### 2.1. Study Design and Participants

Fifteen Black emerging adults aged 18–27 years old completed the SHOW pilot study, described in detail elsewhere [20]. Briefly, this followed a measurement-burst design to investigate associations between habitual, free-living measures of sleep and health outcomes, including appetite sensations and food rewards assessed during an in-laboratory visit over a 6-month period. The participants completed two identical 7-day data collection periods at baseline (BLN) and 6 months later (6MO). All BLN visits were completed between June and November 2022, and all 6MO visits were completed between January and May 2023. For the 7-day study period, the participants wore an ActiGraph GT9X Link accelerometer (ActiGraph Corp., Pensacola, FL, USA) around their wrists and completed daily activity logs to assess habitual sleep. On one day during each 7-day study period, the participants came to the laboratory following an overnight fast and completed anthropometric measures (height, body weight, body composition, and waist circumference), fasting and post-meal appetite measures before and every 30 min for 3 h following standard breakfast intake (540 kcal, 19 g of protein, 76 g of carbohydrate, and 20 g of fat), and a computer-based behavioral choice task to assess measures of food reward prior to receiving an ad libitum lunch. On 2 nights during each 7-day period, the participants were fitted at home with a PSG device to measure habitual sleep, including sleep architecture. The privacy rights of the participants were observed, and written informed consent was obtained from all the participants. The Institutional Review Board at the University of North Carolina at Greensboro approved all the procedures involving human participants (IRB-FY22-433).

### 2.2. Measures

All the measurements were taken at the BLN and 6MO time points. Body height was measured without shoes to the nearest 0.1 cm using a wall-mounted stadiometer (SECA, Chino, CA, USA). Waist circumference was measured with a Gulick tape measure. Body composition and weight were measured to the nearest 0.1% and 0.001 kg, respectively, using air displacement plethysmography (Bod Pod; COSMED, Concord, CA, USA).

Sleep was measured over 7 days using the wrist-worn ActiGraph (Actigraph GTx9 Link, Actigraph LLC, Pensacola, FL, USA), and the Choi algorithm [21] was used to detect non-wear time. Sleep bouts were identified via the Sadeh algorithm [22], which were then visually inspected and compared to the self-reported sleep time reported in the participants’ activity logs. Daily measures of sleep duration (minutes/day) and sleep efficiency (time spent asleep divided by total time spent in bed; %) were averaged for each time point (BLN and 6MO).

The Nox A1 full PSG system (Nox Medical USA, Suwanee, GA, USA) was also used to assess habitual sleep over 2 nights at each time point. This device has demonstrated good feasibility for unsupervised at-home sleep monitoring, compared to in-laboratory sleep monitoring [23]. About 1 h before bedtime, two research assistants traveled to the participants’ homes and equipped them with the PSG device using the 10–20 placement approach for the electrodes [20]. Data collected by the PSG system was downloaded onto the Noxturnal software (US version 6.3.0) for analysis of total sleep duration, sleep efficiency, and sleep stage duration (stage 2, SWS and REM sleep durations; minutes/night) following the American Academy of Sleep Medicine (AASM) criteria [24]. PSG-assessed sleep variables were averaged over the 2 nights at BLN and 6MO. In instances where the participants either woke up after it had stopped recording or fell asleep before the device began monitoring sleep, 1 night of sleep data was used for that time point in the analyses (*n* = 4 nights).

Fasting and post-standard breakfast intake appetite sensations were recorded with 100 mm computerized visual analog scales [25]. The following four questions [26] were asked at every measurement time point: desire to eat (“How strong is your desire to eat?”; very weak—very strong), hunger (“How hungry do you feel?”; Not hungry at all—As hungry as I have ever felt), fullness (“How full do you feel?”; Not full at all—Very full), and prospective food consumption (“How much food do you think you could eat?”; Nothing at all—A large amount). Fasting appetite measures are presented as a single values, whereas post-breakfast values for each appetite measure were combined by calculating the area under the curve (AUC) via the trapezoid method with respect to zero [27].

Measures of food reward were assessed with a computer-based behavioral choice task called the Leeds Food Preference Questionnaire (LFPQ) [28] approximately 3 h following the standard breakfast and prior to an ad libitum lunch. This task measures preference, explicit food liking (i.e., the pleasurable experience of food), and implicit and explicit food wanting (i.e., the motivation to eat, whether conscious [explicit] or non-verbal [implicit]) [29] for 16 different food items that vary in both fat content and taste (high-fat savory, high-fat sweet, low-fat savory, and low-fat sweet) [30]. The 16 food items presented to each participant during this task were chosen by them during a preliminary session to ensure that the food items were familiar and aligned with personal preferences. During the forced choice portion of this task, each food image from a given category was presented with every other food image from a different category in turn. For each pair of food images presented, the participants were asked to select the food they would “most want to eat now” to assess preference. A standardized implicit wanting score for each food item was also calculated as a function of the reaction time in selecting the food item, adjusted for the frequency of choice for food items selected in each category [30]. The participants were also asked to rate the extent to which they “liked” (“How pleasant would it be to experience a mouthful of this food now?”) or “wanted” (“How much do you want to eat this food now?”) each randomly presented food item using a 100 mm visual analog scale to assess explicit liking and explicit wanting, respectively. Fat content and sweet-taste bias scores (i.e., differences between high-fat vs. low-fat scores and sweet vs. savory scores) for each food reward variable were calculated and are presented herein [5,30]. Positive scores indicate a higher preference, liking, or wanting for high-fat or sweet foods, whereas negative scores indicate a higher preference, liking, or wanting for low-fat or savory foods.

### 2.3. Statistical Analyses

Analyses were conducted in R, Version 4.2.2 [31], and statistical significance was set at α < 0.05 considering the exploratory nature of this pilot study. Wilcoxon signed-rank tests were used to evaluate within-person changes in sleep, appetite, and food reward measures between BLN and 6MO. Change scores (calculated as 6MO minus BLN) were calculated and rank-transformed to be included in partial Pearson correlations (fit using the pcor function in the ppcor package in R [32]) assessing the strength of associations between changes in sleep variables with changes in appetite (fasting and AUC values) and food reward outcomes over the 6-month measurement period. BLN body weight and BLN values of relevant sleep predictors were included as covariates.

## 3. Results

A total of 15 participants completed both BLN and 6MO measurement bursts (mean ± SD: BLN age, 21.2 ± 2.4 years; body mass index, 25.7 ± 4.5 kg/m^2^; body fat, 25.8 ± 11.9%). Changes in sleep, appetite, food reward, and body weight variables over 6 months are presented in Table 1. There was a significant increase in body weight (2.6 kg, *p* < 0.01), waist circumference (2.4 cm, *p* = 0.02), and a non-significant change in fat mass (1.9 kg, *p* = 0.09) from BLN to 6MO [20]. Our sample also had a 38 min decrease in actigraph-measured sleep duration (*p* = 0.06) from BLN to 6MO [20]. The mean sleep duration was short (<7 h), and the mean sleep efficiency was poor (<80%) at both BLN (6 h 35 min; 72%) and 6MO (5 h 57 min; 75%). However, PSG-derived sleep duration and sleep efficiency were good (7 h 49 min; 84.5%) and did not differ between the time points [20]. The fasting fullness scores significantly decreased from BLN to 6MO (−8.9, *p* < 0.01). While not reaching statistical significance, the fasting desire to eat (8.3, *p* = 0.06, r = 0.48) and hunger (4.9, *p* = 0.16, r = 0.37) scores increased from BLN to 6MO with moderate effect sizes. There were no changes in the other appetite fasting scores, nor in the post-meal AUC values. Lastly, there were significant increases in explicit liking (11.3, *p* = 0.01), explicit wanting (11.3, *p* = 0.01), implicit wanting (18.4, *p* = 0.02), and preference (6.3, *p* = 0.02) for sweet vs. savory foods from BLN to 6MO. However, there were no significant changes in bias scores for fat content.

### Partial Correlations Between Changes in Sleep with Changes in Appetite and Food Reward

**Figure 1:**
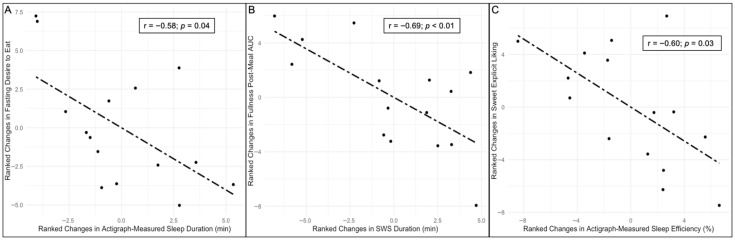
Associations between (**A**) changes in actigraph-measured sleep duration with changes in fasting desire to eat scores, (**B**) changes in SWS duration with changes in post-meal fullness AUC, and (**C**) changes in actigraph-measured sleep efficiency with changes in explicit liking for sweet foods in all participants (*n* = 15). All values are ranked (1–15) and adjusted for baseline body weight and the baseline sleep predictor of interest. A higher-ranked value indicates a greater increase over 6 months.

Table 2 presents the results of the partial Pearson correlations between changes in sleep variables with changes in appetite over 6 months. Changes in actigraph-measured sleep duration were inversely associated with changes in fasting desire to eat scores (r = −0.58, *p* = 0.04), suggesting that increases in habitual sleep duration were associated with decreases in fasting desire to eat over 6 months (Figure 1A). Lastly, changes in SWS duration were inversely associated with post-meal fullness AUC (r = −0.69, *p* < 0.01 [Figure 1B]), suggesting that greater increases in SWS duration were associated with less fullness after a standard meal.

Table 3 presents the results of partial Pearson correlations between changes in sleep variables with changes in food reward. Changes in actigraph-measured sleep efficiency were inversely associated with changes in explicit liking for sweet foods (r = −0.60, *p* = 0.03) (Figure 1C), suggesting that increases in habitual sleep efficiency were associated with greater decreases in explicit liking for sweet foods. There were no other significant associations between sleep and food reward outcomes.

## 4. Discussion

The present paper aimed to investigate whether changes in actigraph- and PSG-measured sleep variables were associated with changes in appetite and food reward over a 6-month period in Black emerging adults. We noted a significant decrease in fasting fullness scores and non-significant increases in fasting desire to eat and hunger scores, despite significant increases in body weight and waist circumference, from BLN to 6MO. We also noted a significant shift in preference, explicit liking, and implicit and explicit wanting for sweet vs. savory foods from BLN to 6MO. While no causal associations can be inferred, these findings suggest that the decreases in fullness ratings and the shift in hedonic preference for sweet foods align with the concurrent decrease in sleep duration and weight gain seen over 6 months in this sample.

Overall, we noted significant inverse associations between actigraph-measured sleep duration and fasting desire to eat scores. This supports our hypothesis and is consistent with previous findings from laboratory-based [33,34,35] and home-based [36] sleep restriction interventions that have reported greater feelings of appetite following imposed sleep restriction. Unlike previous reports from sleep restriction interventions [5,6], we did not observe any associations between REM sleep duration and appetite measures. Contrary to both our hypothesis and to prior evidence [4], we found that increases in SWS duration were associated with decreases in feelings of fullness after a standard meal intake. At both time points, SWS in our sample was higher than previously reported in emerging adults [37] and may be indicative of sleep debt [38] given the high prevalence of short sleepers as confirmed with actigraph-based data in this sample (47% at BLN and 85% at 6MO had on average < 7 h of sleep/day) [20]. Overall, total sleep duration, rather than sleep architecture, may be more strongly associated with reductions in appetite measures, particularly in a sample of emerging adults with predominantly short sleep durations.

Increases in actigraph-measured sleep efficiency were also associated with decreases in explicit liking for sweet foods. This supports similar findings by Kracht et al. [39], who noted an inverse association between actigraph-measured sleep efficiency with cravings for sweet foods in adolescents (ages 10–16) and a second study that reported inverse associations between free-living sleep duration and food reward, specifically food wanting, in young adults [40]. Evidence from home-based sleep restriction studies also reported an increase in sweet preference (as assessed by sucrose solutions) [7] and greater reward for chocolate candies (as measured by a progressive ratio task) [41] after imposed sleep restriction compared to habitual sleep. Overall, it appears that improvements in sleep duration and sleep efficiency may be associated with lower hedonic drive, especially for sweet foods.

To our knowledge, our study is the first to comprehensively assess sleep via actigraphy and PSG under free-living conditions, as well as subjective appetite and food reward measures exclusively in Black emerging adults. This population experiences higher rates of obesity [11,12] and shorter sleep duration [13,14,15,16] compared to peers of other racial groups, which supports a need to better understand mechanisms by which shorter sleep duration and weight gain may occur in this population. We individualized the LFPQ task for each participant to reduce the risk of the participants providing low ratings for items that they are unfamiliar with. Our pilot study also has limitations. Our statistical power is limited by a small sample size, and an uneven distribution of males and females prohibits us from examining sex differences (*n* = 3 males; *n* = 12 females). Menstrual cycle phase was not reported nor accounted for during data collection in the female participants, which may undermine some of our associations or changes in appetite and food reward that vary across the menstrual cycle. Because we only assessed appetite and food reward on one occasion during each time point, we cannot assess the impact of potential day-to-day variability in these measures. However, our in-laboratory protocol that uses a standard breakfast does normalize the environment and conditions during which these measurements were assessed. We did not measure the palatability of the standard breakfast, which may have influenced our appetite findings (i.e., the participants may not regularly consume these items at breakfast or in their normal diet, or may not have found the breakfast to be palatable). If feasible, future studies could consider personalizing the standard breakfast to food preferences measured during a preliminary visit. Despite the noted limitations, this pilot study informed the larger SHOW study [19], which is currently ongoing and will be used to confirm these hypothesis-driven results presented herein in a sample of 150 Black emerging adults over 12 months. We expect that such longitudinal evidence will provide valuable information for the design of targeted sleep interventions, some of which may include specific subgroups of individuals at greatest risk of insufficient sleep, or modify components of sleep most strongly associated with changes in appetite, food reward, and obesity risk.

## 5. Conclusions

We noted decreases in fasting fullness, as well as increases in preference, liking, and wanting for sweet (vs. savory) foods over the 6-month study period, which, along with reductions in sleep duration, may partially contribute to the increase in body weight and waist circumference noted in this sample over the same period. Our results also complement prior research by noting that greater increases in actigraph-measured sleep duration and sleep efficiency were associated with greater decreases in fasting desire to eat and explicit liking for sweet foods, respectively. Future studies with larger sample sizes are needed to confirm our preliminary findings. Indeed, we expect that the SHOW study [19], which aims to recruit 150 Black emerging adults and complete assessments over 12 months, will allow us to further examine and confirm these associations and provide data to inform targeted sleep interventions in this population.

## Figures and Tables

**Table 1:** Changes in body weight, sleep, and appetite and food reward variables from baseline to 6-month follow-up.

	Baseline	6 Months	Difference Between BLN and 6MO
	Mean ± SD	Mean ± SD	Z, *p* Value, r
Anthropometrics			
Body weight (kg) ^†^	72.4 ± 12.6	75.0 ± 12.0	**−3.0, <0.01, 0.76**
Fat mass (kg) ^†^	19.6 ± 11.0	21.5 ± 10.6	−1.70, 0.09, 0.44
Waist circumference (cm) ^†^	77.7 ± 8.0	80.1 ± 7.9	**−2.93, 0.03, 0.76**
Actigraph Sleep Variables			
Sleep duration (min) ^†^	395.4 ± 70.2	357.8 ± 48.5	**1.99, 0.05, 0.51**
Sleep efficiency (%) ^†^	72.6 ± 7.5	75.8 ± 7.8	−1.76, 0.08, 0.45
PSG Sleep Variables			
Sleep duration (min) ^†^	469.9 ± 68.2	463.5 ± 52.8	0.51, 0.63, 0.13
Sleep efficiency (%) ^†^	84.5 ± 14.6	88.7 ± 8.7	−0.85, 0.41, 0.22
REM duration (min) ^†^	75.6 ± 35.4	83.8 ± 42.1	−1.08, 0.49, 0.28
N2 duration (min) ^†^	284.0 ± 62.0	260.0 ± 60.0	1.65, 0.11, 0.43
N3 duration (min) ^†^	104.7 ± 30.6	104.7 ± 27.0	0.06, 0.98, 0.02
Appetite Scores			
Fasting desire to eat	59.0 ± 35.0	67.3 ± 38.6	−1.87, 0.06, 0.48
Desire to eat post-meal AUC	8260.3 ± 3784.7	8596.3 ± 3262.3	−0.28, 0.80, 0.07
Fasting hunger	53.4 ± 28.3	58.3 ± 31.0	−1.42, 0.16, 0.37
Hunger post-meal AUC	7045.0 ± 3253.9	7865 ± 2856.8	−0.97, 0.35, 0.25
Fasting fullness	28.5 ± 28.9	19.6 ± 27.2	**2.02, <0.01, 0.52**
Fullness post-meal AUC	8198.0 ± 2529.1	8341.5 ± 3013.2	0.57, 0.59, 0.15
Fasting prospective food consumption	58.4 ± 24.3	61.3 ± 32.0	−0.85, 0.41, 0.22
Prospective food consumption post-meal AUC	7958.1 ± 2650.6	8542.5 ± 3378.9	−0.68, 0.51, 0.18
Food Reward			
Fat—explicit liking	−9.1 ± 18.6	−11.0 ± 14.6	0.74, 0.48, 0.19
Fat—explicit wanting	−11.6 ± 18.0	−10.4 ± 14.4	−0.03, 1.00, 0.01
Fat—implicit wanting	−18.3 ± 35.0	−17.3 ± 31.5	−0.34, 0.75, 0.09
Fat preference	−5.8 ± 12.1	−5.7 ± 11.3	−0.20, 0.86, 0.05
Sweet—explicit liking	−7.3 ± 23.1	4.0 ± 14.8	**−2.56, 0.01, 0.66**
Sweet—explicit wanting	−8.5 ± 23.0	2.8 ± 17.6	**−2.56, 0.01, 0.66**
Sweet—implicit wanting	−17.8 ± 41.3	0.6 ± 30.6	**−2.33, 0.02, 0.60**
Sweet preference	−5.8 ± 12.7	0.5 ± 11.1	**−2.78, 0.02, 0.72**

Changes in sleep and appetite and food reward variables from baseline to 6-month follow-up (*n* = 15). Mean difference (MD) is calculated as 6MO-BLN; a positive value indicates an increase at 6MO. Appeal bias scores were calculated for each endpoint of the LFPQ (preference, liking, and wanting), for fat (high vs. low fat), and taste (sweet vs. savory). Positive scores indicate a higher preference, liking, or wanting for high-fat or sweet foods, whereas negative scores indicate a higher preference, liking, or wanting for low-fat or savory foods. Bold type indicates a *p* < 0.05. ^†^ Results are presented elsewhere [20].

**Table 2:** Results from partial Pearson correlations between changes in sleep variables with changes in appetite measures.

Sleep Variable	Coefficient (r)	*p*-Value
Actigraph—Total sleep duration (min)		
Fasting desire to eat	**−0.58**	**0.04**
Desire to eat post-meal AUC	−0.05	0.87
Fasting hunger	−0.47	0.11
Hunger post-meal AUC	0.03	0.93
Fasting fullness	0.34	0.26
Fullness post-meal AUC	−0.19	0.54
Fasting prospective food consumption	−0.44	0.13
Prospective food consumption post-meal AUC	0.15	0.63
Actigraph—Sleep efficiency (%)		
Fasting desire to eat	0.34	0.25
Desire to eat post-meal AUC	0.07	0.83
Fasting hunger	−0.10	0.75
Hunger post-meal AUC	−0.01	0.97
Fasting fullness	0.08	0.78
Fullness post-meal AUC	−0.27	0.38
Fasting prospective food consumption	0.42	0.15
Prospective food consumption post-meal AUC	0.37	0.22
Polysomnography—Total sleep duration (min)		
Fasting desire to eat	−0.26	0.40
Desire to eat post-meal AUC	−0.26	0.40
Fasting hunger	−0.08	0.79
Hunger post-meal AUC	−0.09	0.77
Fasting fullness	0.18	0.56
Fullness post-meal AUC	0.36	0.23
Fasting prospective food consumption	−0.37	0.21
Prospective food consumption post-meal AUC	−0.33	0.28
Polysomnography—Sleep efficiency (%)		
Fasting desire to eat	0.05	0.87
Desire to eat post-meal AUC	−0.14	0.64
Fasting hunger	−0.18	0.56
Hunger post-meal AUC	0.05	0.88
Fasting fullness	−0.03	0.93
Fullness post-meal AUC	−0.05	0.87
Fasting prospective food consumption	0.01	0.98
Prospective food consumption post-meal AUC	0.04	0.91
Stage 2 sleep duration (min)		
Fasting desire to eat	−0.10	0.75
Desire to eat post-meal AUC	−0.08	0.80
Fasting hunger	−0.21	0.48
Hunger post-meal AUC	0.09	0.78
Fasting fullness	0.07	0.82
Fullness post-meal AUC	0.01	0.98
Fasting prospective food consumption	0.27	0.36
Prospective food consumption post-meal AUC	0.02	0.96
Slow-wave sleep duration (min)		
Fasting desire to eat	−0.03	0.91
Desire to eat post-meal AUC	0.05	0.88
Fasting hunger	−0.26	0.39
Hunger post-meal AUC	0.40	0.17
Fasting fullness	−0.45	0.13
Fullness post-meal AUC	**−0.69**	**<0.01**
Fasting prospective food consumption	−0.43	0.15
Prospective food consumption post-meal AUC	0.19	0.53
Rapid eye movement sleep duration (min)		
Fasting desire to eat	−0.33	0.28
Desire to eat post-meal AUC	−0.06	0.85
Fasting hunger	0.23	0.45
Hunger post-meal AUC	−0.10	0.75
Fasting fullness	0.16	0.61
Fullness post-meal AUC	0.49	0.09
Fasting prospective food consumption	−0.36	0.23
Prospective food consumption post-meal AUC	−0.26	0.39

Results from partial Pearson correlations between changes in actigraphy- and polysomnography-based sleep variables with changes in appetite measures across 6 months. Covariates included the baseline value of the sleep predictor of interest and baseline body weight. Bold type indicates statistical significance.

**Table 3:** Results from partial Pearson correlations between changes in sleep variables with changes in food reward.

Sleep Variable	Coefficient (r)	*p*-Value
Actigraphy—Total sleep duration (min)		
Fat explicit liking	0.42	0.15
Fat explicit wanting	0.55	0.05
Fat implicit wanting	0.42	0.15
Fat preference	0.48	0.09
Sweet explicit liking	0.43	0.15
Sweet explicit wanting	0.36	0.22
Sweet implicit wanting	0.44	0.13
Sweet preference	0.43	0.14
Actigraphy—Sleep efficiency (%)		
Fat explicit liking	−0.36	0.23
Fat explicit wanting	−0.28	0.35
Fat implicit wanting	−0.39	0.19
Fat preference	−0.30	0.32
Sweet explicit liking	**−0.60**	**0.03**
Sweet explicit wanting	−0.14	0.65
Sweet implicit wanting	−0.18	0.55
Sweet preference	−0.41	0.16
Polysomnography—Total sleep duration (min)		
Fat explicit liking	0.23	0.45
Fat explicit wanting	0.44	0.13
Fat implicit wanting	0.15	0.63
Fat preference	0.03	0.91
Sweet explicit liking	0.43	0.14
Sweet explicit wanting	0.07	0.83
Sweet implicit wanting	−0.13	0.67
Sweet preference	0.20	0.52
Polysomnography—Sleep efficiency (%)		
Fat explicit liking	0.09	0.77
Fat explicit wanting	0.24	0.42
Fat implicit wanting	0.03	0.93
Fat preference	0.16	0.59
Sweet explicit liking	0.22	0.47
Sweet explicit wanting	0.51	0.08
Sweet implicit wanting	0.08	0.80
Sweet preference	−0.05	0.87
Stage 2 sleep duration (min)		
Fat explicit liking	−0.07	0.81
Fat explicit wanting	0.05	0.86
Fat implicit wanting	−0.07	0.82
Fat preference	−0.20	0.52
Sweet explicit liking	−0.03	0.91
Sweet explicit wanting	0.00	1.00
Sweet implicit wanting	−0.21	0.50
Sweet preference	0.01	0.97
Slow-wave sleep duration (min)		
Fat explicit liking	0.32	0.28
Fat explicit wanting	0.39	0.19
Fat implicit wanting	0.14	0.65
Fat preference	0.15	0.62
Sweet explicit liking	0.31	0.31
Sweet explicit wanting	0.30	0.32
Sweet implicit wanting	0.51	0.08
Sweet preference	0.52	0.07
Rapid eye movement sleep duration (min)		
Fat explicit liking	0.08	0.79
Fat explicit wanting	0.16	0.61
Fat implicit wanting	0.18	0.55
Fat preference	0.17	0.59
Sweet explicit liking	0.36	0.23
Sweet explicit wanting	−0.14	0.64
Sweet implicit wanting	−0.18	0.57
Sweet preference	0.00	1.00

Results from partial Pearson correlations between changes in actigraphy- and polysomnography-based sleep variables with changes in food reward across 6 months. Covariates included the baseline value of the sleep predictor of interest and baseline body weight. Bold type indicates statistical significance.

## Data Availability

The data that supports these findings are openly available in the OSF repository at https://osf.io/tqsmj/.
